# IL-10 production by granulocytes promotes *Staphylococcus aureus* craniotomy infection

**DOI:** 10.1186/s12974-023-02798-7

**Published:** 2023-05-13

**Authors:** Gunjan Kak, Zachary Van Roy, Cortney E. Heim, Rachel W. Fallet, Wen Shi, Axel Roers, Bin Duan, Tammy Kielian

**Affiliations:** 1 Department of Pathology and Microbiology, University of Nebraska Medical Center, 985900 Nebraska Medical Center, Omaha, NE 68198-5900 USA; 2 Mary and Dick Holland Regenerative Medicine Program, Division of Cardiology, Department of Internal Medicine, University of Nebraska Medical Center, Omaha, NE USA; 3 Institute of Immunology, Heidelberg University Hospital, Heidelberg, Germany

**Keywords:** Interleukin-10, Microglia, Granulocytes, Craniotomy infection, *S. aureus*

## Abstract

**Background:**

Treatment of brain tumors, epilepsy, or hemodynamic abnormalities requires a craniotomy to access the brain. Nearly 1 million craniotomies are performed in the US annually, which increase to ~ 14 million worldwide and despite prophylaxis, infectious complications after craniotomy range from 1 to 3%. Approximately half are caused by *Staphylococcus aureus* (*S. aureus*), which forms a biofilm on the bone flap that is recalcitrant to antibiotics and immune-mediated clearance. However, the mechanisms responsible for the persistence of craniotomy infection remain largely unknown. The current study examined the role of IL-10 in promoting bacterial survival.

**Methods:**

A mouse model of *S. aureus* craniotomy infection was used with wild type (WT), IL-10 knockout (KO), and IL-10 conditional KO mice where IL-10 was absent in microglia and monocytes/macrophages (*CX3CR1*^*Cre*^*IL-10*^* fl/fl*^) or neutrophils and granulocytic myeloid-derived suppressor cells (G-MDSCs; *Mrp8*^*Cre*^*IL-10*^* fl/fl*^), the major immune cell populations in the infected brain vs. subcutaneous galea, respectively. Mice were examined at various intervals post-infection to quantify bacterial burden, leukocyte recruitment, and inflammatory mediator production in the brain and galea to assess the role of IL-10 in craniotomy persistence. In addition, the role of G-MDSC-derived IL-10 on neutrophil activity was examined.

**Results:**

Granulocytes (neutrophils and G-MDSCs) were the major producers of IL-10 during craniotomy infection. Bacterial burden was significantly reduced in IL-10 KO mice in the brain and galea at day 14 post-infection compared to WT animals, concomitant with increased CD4^+^ and γδ T cell recruitment and cytokine/chemokine production, indicative of a heightened proinflammatory response. *S. aureus* burden was reduced in *Mrp8*^*Cre*^*IL-10*^* fl/fl*^ but not *CX3CR1*^*Cre*^*IL-10*^* fl/fl*^ mice that was reversed following treatment with exogenous IL-10, suggesting that granulocyte-derived IL-10 was important for promoting *S. aureus* craniotomy infection. This was likely due, in part, to IL-10 production by G-MDSCs that inhibited neutrophil bactericidal activity and TNF production.

**Conclusion:**

Collectively, these findings reveal a novel role for granulocyte-derived IL-10 in suppressing *S. aureus* clearance during craniotomy infection, which is one mechanism to account for biofilm persistence.

**Supplementary Information:**

The online version contains supplementary material available at 10.1186/s12974-023-02798-7.

## Introduction

Craniotomy is a neurosurgical procedure required to access the brain for the treatment of tumors, epilepsy, or hemodynamic abnormalities [1]. Nearly 1 million craniotomies are performed in the US annually, which increases to ~ 14 million worldwide. Despite prophylaxis, infectious complications manifest in approximately 1–3% of craniotomies and can reach upwards of 15% [2]. The Gram-positive pathogen *Staphylococcus aureus* (*S. aureus*) is responsible for half of craniotomy infections [3, 4], where treatment is complicated by the ability of *S. aureus* to form biofilm, a lifestyle that renders bacteria recalcitrant to antibiotics [5]. This arises, in part, from the metabolic dormancy of a subpopulation of bacteria, termed persister cells, that are tolerant to antibiotics that target active protein and cell wall biosynthesis [6]. Craniotomy infection typically necessitates a second neurosurgery to debride the affected tissue; however, in many cases the infected bone flap is discarded out of concern for residual biofilm that can re-establish infection [7–9]. This highlights the importance of understanding the factors that are responsible for promoting biofilm formation and persistence to devise novel therapeutic approaches to reduce the morbidity associated with craniotomy infection.

The balance between pro- and anti-inflammatory pathways is critical for clearing infection and resolving inflammation. This is particularly important in the CNS because of the potential for bystander damage to surrounding normal brain parenchyma from a dysregulated proinflammatory response, which is compounded by the inability of neurons to regenerate to an appreciable extent. Our prior work has established the existence of proinflammatory responses that are critical for preventing *S. aureus* outgrowth during craniotomy infection; particularly the Toll-like receptor 2 (TLR2)-IL-1β axis and neutrophil action [10, 11]. However, phenotypes are only manifested in the absence of these proinflammatory pathways, demonstrating their inability to clear infection in the wild type setting and that anti-inflammatory mechanisms outweigh these responses.

One such candidate is IL-10, which functions as a secreted homodimer that binds to the IL-10R, a heterodimeric receptor composed of a unique IL-10Rα subunit and an IL-10Rβ subunit that is shared by other IL-10 family members [12]. Upon engagement of the receptor, IL-10 signaling leads to signal transducer and activator of transcription 3 (STAT3) activation and the expression of STAT3-dependent target genes [13, 14]. IL-10 classically functions as an anti-inflammatory cytokine, where it inhibits macrophage activation, cytokine production, and metabolism [15–19]. Within the CNS, IL-10 is produced by resident microglia and astrocytes as well as infiltrating leukocytes during neuroinflammatory diseases [20, 21] and IL-10 production is elicited in response to TLR2 signaling during *S. aureus* craniotomy infection [10]. Despite its well-known anti-inflammatory effects, IL-10 can also exert paradoxical proinflammatory activity [14]. The most well-characterized proinflammatory action of IL-10 is via potentiating IFN-ɣ and granzyme B production by activated CD8^+^ T cells, and exogenous IL-10 has been reported to increase systemic IFN-ɣ levels in humans [22–26]. However, there are few CD8^+^ T cell infiltrates during *S. aureus* craniotomy infection [11], suggesting that the actions of IL-10 in this setting are likely anti-inflammatory due to the predominance of resident microglia and infiltrating myeloid cells.

Various T cell subsets have been shown to play essential roles during *S. aureus* skin and soft tissue infections, where CD4^+^ and ɣδ T cells are critical for infection containment [27–29]. However, it is noteworthy that both populations are absent from the galea during *S. aureus* craniotomy infection [11, 30], which is in direct contact with the scalp, revealing a unique cutaneous response to infection that may be driven by biofilm formation. In mouse models of peripheral infection, IL-10 was critical for promoting *S. aureus* skin infection where IL-10 KO mice had lower bacterial burden concomitant with increased phagocyte, ɣδ T cell, and CD4^+^ T cell recruitment [31]. IL-10 is also important for *S. aureus* persistence during prosthetic joint infection, which was mediated by IL-10 produced by infiltrating granulocytic myeloid-derived suppressor cells (G-MDSCs) and monocytes/macrophages [32, 33]. Furthermore, *S. aureus* metabolites are known to elicit IL-10 production that can manipulate protective immune responses by triggering epigenetic changes to ensure long-term bacterial survival within the host [34].

However, since IL-10 is produced by a wide array of leukocytes as well as resident CNS glia [14, 20], it was of interest to explore the functional importance of IL-10 during craniotomy infection and identify the key sources of the cytokine using IL-10 conditional KO mice. IL-10 production was critical for *S. aureus* persistence during craniotomy infection, as evidenced by the significant reduction in bacterial burden in the brain and galea of IL-10 KO mice compared to WT animals. Decreased bacterial abundance coincided with enhanced cytokine/chemokine production and CD4^+^ and ɣδ T cell recruitment in the brain. An important role for granulocyte-derived IL-10 in promoting *S. aureus* craniotomy infection was established using *Mrp8*^*Cre*^*IL-10*^* fl/fl*^ mice that displayed decreased bacterial burden that was likely due, in part, to IL-10 production by G-MDSCs that inhibited polymorphonuclear neutrophil (PMN) bactericidal activity and TNF expression. Treatment of *Mrp8*^*Cre*^*IL-10*^* fl/fl*^ mice with IL-10 microparticles increased *S. aureus* burden compared to empty microparticles, confirming the critical role of IL-10 in promoting *S. aureus* survival during craniotomy infection. However, phenotypes with *Mrp8*^*Cre*^*IL-10*^* fl/fl*^ mice were less dramatic compared to IL-10 KO animals, suggesting the collaborative action of multiple sources of IL-10 to attenuate proinflammatory responses and facilitate *S. aureus* craniotomy persistence.

## Materials and methods

### Mouse strains

IL-10 KO mice were obtained from The Jackson Laboratory (RRID:IMSR_JAX:002251) with C57BL/6J animals (RRID:IMSR_JAX:000664) used as WT controls. IL-10 floxed mice were generated as previously described [35] and crossed to R26-stop-EYFP mice (RRID:IMSR_JAX:006148) where the Enhanced Yellow Fluorescent Protein gene (referred to as YFP throughout the manuscript) is flanked by a *loxP* stop sequence to identify cells where the *Il-10* gene had been deleted by Cre-mediated excision. *CX3CR1*^*Cre*^ (RRID:IMSR_JAX:025524) and *Mpr8*^*Cre*^-GFP (RRID:IMSR_JAX:021614) mice were crossed to IL-10/R26-EYFP animals to delete *Il-10* in microglia and macrophages vs. PMNs and G-MDSCs, respectively. In our hands, the GFP signal in *Mpr8*^*Cre*^-GFP mice was weak; therefore, *Il-10* excision was marked by YFP in both strains. Wild type littermates were used as controls for both *CX3CR1*^*Cre*^*IL-10*^* fl/fl*^ and *Mpr8*^*Cre*^*IL-10*^* fl/fl*^ mice. Animals were bred at the University of Nebraska Medical Center (UNMC) vivarium in a high-SPF barrier area and mice of the same sex were randomized into standard density cages upon weaning. Upon *S. aureus* infection, mice were housed in a restricted-access BSL2 room equipped with ventilated microisolator cages and maintained at 21 °C under a 12 h light:12 h dark cycle with ad libitum access to water (Hydropac™; Lab Products, Seaford, DE) and Teklad rodent chow (Harlan, Indianapolis, IN) with Nestlets provided for enrichment.

### Mouse model of S. aureus craniotomy infection

*S. aureus* craniotomy infection was established in 8- to 10-week-old male and female mice as previously described, where biofilm formation on the bone flap establishes persistent infection in the subcutaneous galea and brain [10, 11, 36, 37]. Briefly, following ketamine/xylazine anesthesia, a skin incision was made to expose the skull and a high-speed pneumatic drill was used to create a bone flap (approximately 3–5 mm in diameter) with care taken to minimize damage to the dura. The excised bone flap was incubated with *S. aureus* strain USA300 LAC13c [38] for 5 min to allow for bacterial adherence (10^3^ colony forming units (CFUs) per bone flap), rinsed with 1X phosphate buffered saline (PBS), and immediately reinserted into the skull, whereupon the skin incision was closed with sutures. Mice received buprenorphine slow release for pain relief during the first 72 h following surgery, were monitored daily, and euthanized at the indicated time points for experimental assessments. No mortality or discernable weight loss was observed throughout the course of experiments in either WT mice or the various IL-10-deficient models.

### Tissue collection and processing for bacterial quantification

Mice were euthanized using an overdose of inhaled isoflurane at the indicated intervals post-infection and the vasculature was immediately perfused with PBS. The bone flap was removed first and vortexed in PBS for 30 s to recover loosely adherent leukocytes, followed by sonication for 5 min to dislodge biofilm-associated bacteria. The galea, representing the subcutaneous tissue and purulent exudate, was dissociated in 1X PBS using the blunt end of a plunger from a 3-cc syringe. Finally, the ipsilateral brain hemisphere underlying the infected bone flap was removed and homogenized by pressing through a 70-µm cell strainer and rinsed with 1X PBS. Once all tissues were processed, aliquots were removed to quantify bacterial burden by plating serial dilutions on tryptic soy agar (TSA) plates supplemented with 5% sheep blood, which are expressed as log_10_ CFU.

### Flow cytometry and intracellular cytokine staining

Following the removal of aliquots from tissue homogenates for quantifying *S. aureus* burden as described above, leukocyte infiltrates in the brain, galea, and bone flap were assessed by flow cytometry. Brain homogenates required additional processing to yield a single-cell suspension. Briefly, brain tissue was incubated in HBSS containing collagenase IV and DNase I for 15 min at 37 °C, whereupon enzymatic activity was inactivated with fetal bovine serum (FBS). Cells were added to a 25% Percoll solution containing 3% FBS and centrifuged at 520x*g* for 20 min with no brake. The upper myelin layer down to the pellet was discarded, and the pellet was resuspended in 1X PBS. Cells from the brain, galea, and bone flap were passed through a filter top FACS tube and incubated with TruStain FcX (BioLegend) to block non-specific antibody binding followed by staining with an innate immune panel that included CD45-BUV805 (Cat. #748370; BD Biosciences) or CD45-APC (RRID:AB_312977), Ly6G-BUV395 (RRID:AB_2739417) or Ly6G-PE (RRID:AB_1186099), CX3CR1-BV785 (RRID:AB_2632858), CD11b-BV570 (RRID:AB_10896949) or CD11b-FITC (RRID:AB_312789), F4/80-BV510 (RRID:AB_2562622) or F4/80-PECy7 (RRID:AB_893490), and Ly6C-APC Cy7 (Cat. #560596; BD Biosciences) or Ly6C-PerCP-Cy5.5 (Cat. #560525 BD). The brain was also stained with an adaptive immune panel that included CD45-BUV805 (Cat. #748370; BD Biosciences) or CD45-APC (RRID:AB_312977), CD3ε-BUV737 (Cat. #741788, BD Biosciences), CD4-PacificBlue (RRID:AB_493374), CD8a-BV605 (RRID:AB_2562609), ɣδTCR-PE (RRID:AB_313832) or ɣδTCR-BV510 (AB_2563534), and NK1.1-APC-Cy7 (RRID:AB_830871).

For intracellular cytokine staining of T cell populations, single-cell suspensions from the brain of each mouse were pooled and subjected to CD3^+^ cell enrichment using a MojoSort™ Mouse CD3 Selection Kit (Cat. #480031; BioLegend) per the manufacturer’s instructions. Purified CD3^+^ cells were treated immediately ex vivo with cell stimulation cocktail (PMA and ionomycin with brefeldin A) for 4 h, and incubated with TruStain FcX for 10 min on ice prior to surface staining with CD45-AF700 (RRID: AB_493715), CD3-Pacific Blue (RRID:AB_493645) or CD3-FITC (RRID:AB_312660), CD4-BV650 (RRID:AB_2562529), and ɣδTCR-BV510 (RRID:AB_2563534). Next, cells were treated with a Cyto-Fast Fix/Perm Buffer Set (Cat. #426803; BioLegend), whereupon intracellular cytokine staining was performed using IFN-γ-APC (RRID:AB_315403), IL-17A-PE (RRID:AB_315463), IL-10-PECy7 (RRID:AB_11150582), and TNF-PerCP-Cy5.5 (RRID:AB_961434).

To identify which innate immune cell populations were the main producers of IL-10 in the infected brain and galea, cell suspensions from each animal were treated immediately ex vivo with brefeldin A for 4 h. Next, cells were incubated with TruStain FcX for 10 min on ice followed by staining with the innate surface marker panel described above, whereupon IL-10 was detected by intracellular staining using IL-10-PECy7 (RRID:AB_11150582). For all analyses, dead cells were excluded using a Zombie UV™ Fixable Viability Kit (Cat. #423108; BioLegend) and analysis was performed on a BD Fortessa cytometer and analyzed with FlowJo (RRID:SCR_008520) using the gating strategy presented in Additional file 1: Fig. S1.

### Quantification of inflammatory mediator expression

Brain and galea tissues from infected mice were processed as described above, whereupon inflammatory mediator expression in cell-free homogenates was measured using Milliplex multi-analyte bead arrays (Cat. #MCYTMAG70PMX25BK; Milliplex, MilliporeSigma). In some experiments, IL-10 was quantified by ELISA (Mouse IL-10 DuoSet ELISA Cat. #DY417-05). Values were normalized to total protein to correct for differences in tissue sampling size.

Cells recovered from brain and galea homogenates of infected WT and IL-10 KO mice were cultured immediately ex vivo in a 48-well plate at 10^5^ cells/well in antibiotic-containing medium (penicillin/streptomycin), whereupon supernatants were collected at 24 h to quantify inflammatory mediator production using a mouse inflammation CBA kit (Cat. #552364, BD Biosciences) according to the manufacturer’s instructions.

### Synthesis of IL-10 microparticles and in vivo administration

To demonstrate the functional importance of IL-10 during *S. aureus* craniotomy infection, animals received IL-10 containing microparticles to provide a continual source of cytokine. This approach had the added benefits of local delivery to the site of infection combined with slow release to modulate the immune response. IL-10 loaded poly(lactide-co-glycolide) (PLGA) microparticles were prepared as previously described with BSA loaded microparticles used as a control [10]. Prior to in vivo use, the kinetics of IL-10 release from microparticles in vitro was monitored by ELISA (Mouse IL-10 DuoSet ELISA Cat. #DY417-05), which revealed sustained cytokine release over 14 days (Additional file 1: Fig. S2). For in vivo experiments, a total dose of 100 ng of IL-10 was administered to each mouse that included both encapsulated and free cytokine. The free IL-10 provided an immediate source of cytokine, while the PLGA microparticles provided long-term IL-10 release, an approach that we successfully used in a prior study for IL-1β delivery [10]. A total of 5 µl of microparticles were applied at both the ventral and dorsal aspects of the bone flap at the time of infection (~ 50 ng loading dose per location). This approach ensured that both surfaces of the bone flap were exposed to IL-10 to modulate inflammatory responses in the brain and galea. Mice were euthanized at day 14 post-infection to quantify IL-10 effects on bacterial burden and leukocyte infiltrates.

### Antibody-mediated depletion of T cell populations

The potential role of CD4^+^ and ɣδ T cells during *S. aureus* craniotomy infection in IL-10 KO mice was assessed by antibody-mediated depletion as previously described with minor modifications [39, 40]. Briefly, animals received i.p. injections of InVivoMab anti-mouse CD4 (Cat. #BE0003-1), InVivoMab anti-mouse ɣδ TCR (Cat. #BE0070), or appropriate isotype-matched control antibodies (300 µg/mouse; all from BioXCell) beginning two to three days prior to *S. aureus* challenge, with repeat injections every 3 or 4 days (for ɣδ and CD4 T cell depletion, respectively) until animals were euthanized at day 14 post-infection. Cell depletion was confirmed by flow cytometry.

### Effects of G-MDSCs on PMN functional activity

To examine the effect of G-MDSC-derived IL-10 on PMN bactericidal activity, a gentamicin protection assay was performed [11]. Mouse bone marrow-derived G-MDSCs were prepared as previously described [41] and PMNs were recovered from the peritoneal cavity by lavage with 1X PBS 24 h after injection of 4% sterile thioglycolate broth, whereupon both populations were purified using anti-Ly6G MicroBeads (Miltenyi Biotec). WT PMNs were added to a 24-well plate at 3 × 10^5^ cells/well and co-cultured for 2 h with 9 × 10^5^ WT or IL-10 KO G-MDSCs that were separated by a 0.4 µm Transwell insert. This 3:1 ratio (G-MDSC:PMN) was used to model cell abundance in the galea during craniotomy infection [11]. Following the 2 h conditioning period, the G-MDSCs in Transwells were removed and held in culture medium. At that point, PMNs were challenged with live *S. aureus* USA300 LAC13c [38] at an MOI of 10:1 (bacteria:PMN) for 2 h. Next, PMNs were centrifuged, washed, and treated with 100 µg/mL gentamicin for 30 min to kill residual extracellular bacteria, whereupon fresh medium containing 1 µg/mL gentamicin was added. Then the G-MDSCs in Transwell inserts were re-introduced with PMNs, and intracellular bacterial burden in PMNs was determined at time 0 (following high-dose gentamicin treatment) and 2 h by lysing cells in H_2_O. In other experiments, the bactericidal activity of PMNs recovered from the bone marrow of *Mrp8*^*Cre*^*IL-10*^* fl/fl*^ and *CX3CR1*^*Cre*^*IL-10*^* fl/fl*^ mice was performed by gentamicin protection assays as described above.

To examine how G-MDSCs modulated PMN activation and the role of IL-10 in this process, a similar co-culture paradigm was performed with PMNs ± WT or IL-10 KO G-MDSCs, whereupon PMN H_2_O_2_ and TNF production was examined. Briefly, after a 30 min or 2 h co-culture period with G-MDSCs, PMNs were stained with OxiVision (Cat. #11505; AAT Bioquest) to quantify H_2_O_2_ production and Zombie UV™ Fixable Viability Dye to exclude dead cells. TNF staining was performed on PMNs following a 30 min co-culture interval with WT or IL-10 KO G-MDSCs, whereupon PMNs were treated with brefeldin A for 4 h followed by viability staining with Zombie UV™ Fixable Viability Dye. PMNs were then fixed and permeabilized followed by intracellular staining with TNF-PerCP-Cy5.5 (RRID:AB_961434). In separate experiments, cytokine production was assessed in PMNs isolated from the bone marrow of *Mrp8*^*Cre*^*IL-10*^* fl/fl*^ and *CX3CR1*^*Cre*^*IL-10*^* fl/fl*^ mice following *S. aureus* exposure by intracellular cytokine staining with IL-6-APC (RRID: AB_504508) and TNF-PerCP-Cy5.5 (RRID:AB_961434). All analyses were performed using a BD Fortessa cytometer and FlowJo as described above.

### Statistics

Significant differences between treatment groups at a given time point were determined as indicated using an unpaired two-tailed Student’s *t*-test, one-way ANOVA, or two-way ANOVA with Tukey’s correction using GraphPad Prism (RRID:SCR_002798). For quantification of inflammatory mediator expression by Milliplex arrays, outliers were identified by the ROUT method [42] (with *Q* = 1%) using GraphPad Prism. Data were tested for normality using Shapiro–Wilk and Kolmogorov–Smirnov tests. Non-normally distributed data were analyzed using a Mann–Whitney *U *test or a Kruskal–Wallis test with Dunn’s multiple comparison when comparing more than two groups. For all analysis, a *p*-value < 0.05 was considered statistically significant.

## Results

### IL-10 is important for regulating T cell recruitment and inflammatory mediator production during *S. aureus* craniotomy infection

**Fig. 1:**
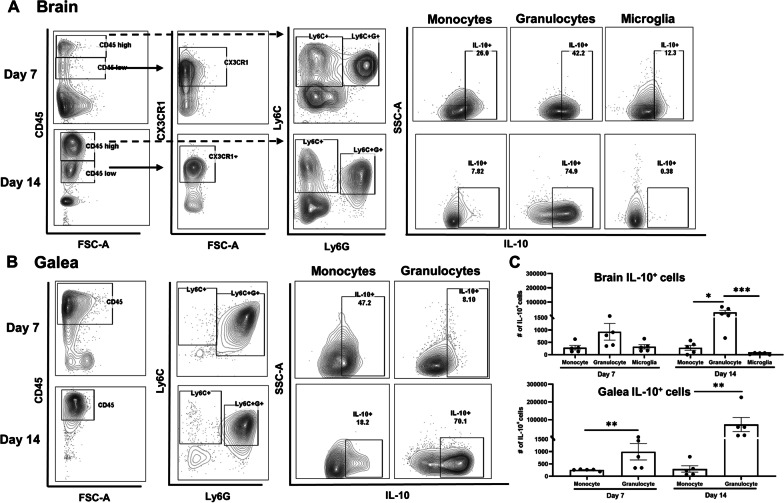
Granulocytes are the major source of IL-10 during *S. aureus* craniotomy infection. Cells were recovered from the **A** brain and **B** galea at days 7 and 14 post-infection and treated immediately ex vivo with brefeldin A (5 µg/mL) for 4 h. Cells were stained with antibodies for CD45, Ly6C, Ly6C, and CX3CR1 to identify granulocytes (PMNs and G-MDSCs; CD45^hi^Ly6G^+^Ly6C^+^), monocytes (CD45^hi^Ly6G^−^Ly6C^+^), and microglia (CD45^lo^CX3CR1^+^), and subsequently fixed and permeabilized to evaluate IL-10 production by intracellular cytokine staining. **C** The absolute numbers of IL-10^+^ cells for each cell type are reported (mean ± SD from *n* = 5 mice/group for each time point; *p < 0.05; **p < 0.01; ***p < 0.01; Brain samples were analyzed using a Kruskal–Wallis test with Dunn’s multiple comparison and differences between galea samples were analyzed with a Mann–Whitney *U* test. The brain and galea samples for each time point were processed on different days and analyzed using separate gates with appropriate compensation and negative controls

**Fig. 2:**
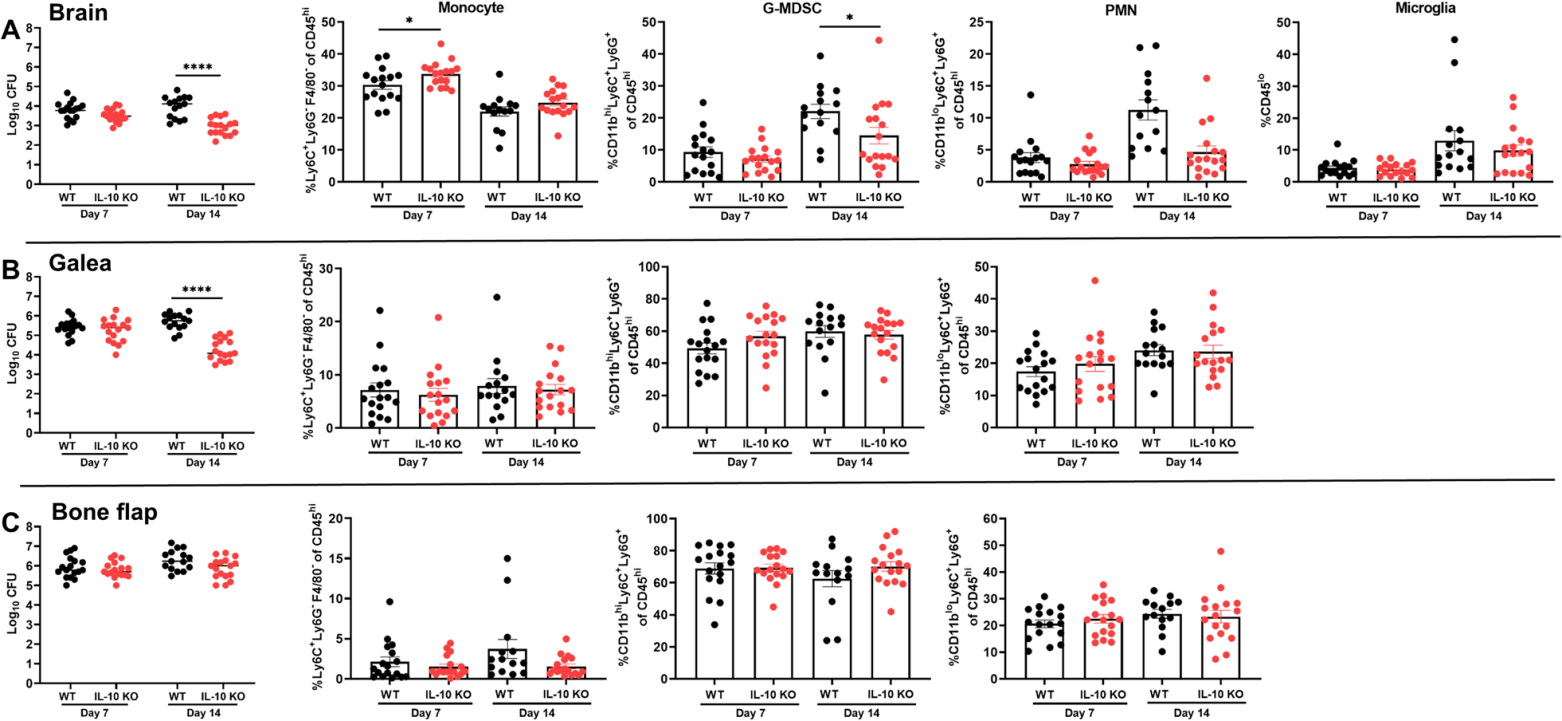
IL-10 is important for promoting *S. aureus* craniotomy infection. IL-10 knockout (KO; *n* = 17) and wild type (WT; *n* = 15–17) mice were euthanized at the indicated time points following *S. aureus* craniotomy infection, whereupon bacterial burden and the percentages of monocyte, granulocytic myeloid-derived suppressor cell (G-MDSC), and neutrophil (PMN) infiltrates were quantified in the **A** brain along with resident microglia, **B** galea, and **C** bone flap. Results are combined from three independent experiments (mean ± SEM) and significant differences between IL-10 KO and WT mice are denoted by asterisks (*p < 0.05; ****p < 0.0001; unpaired Student’s *t*-test)

**Fig. 3:**
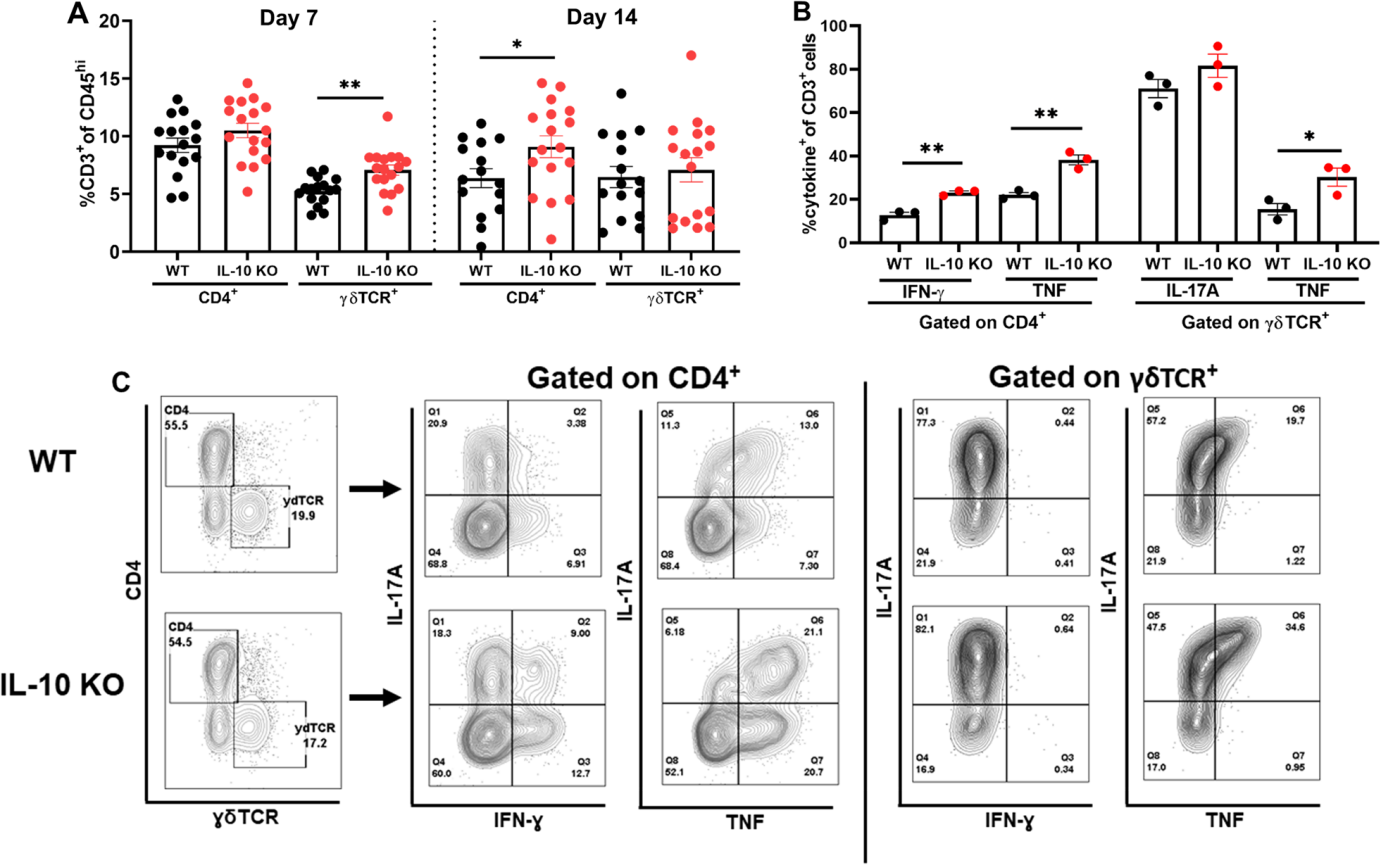
IL-10 loss results in an increased proinflammatory signature in T cells infiltrating the infected brain. **A** Quantification of CD4^+^ and ɣδ T cell infiltrates in the brain of IL-10 knockout (KO; *n* = 17) and wild type (WT; *n* = 15–16) mice at days 7 and 14 post-infection. Results are combined from three independent experiments (mean ± SEM) and significant differences between IL-10 KO and WT mice are denoted by asterisks (*p < 0.05; **p < 0.01; unpaired Student’s *t*-test). In **B** and **C**, CD3 cells were isolated from the brains of WT and IL-10 KO animals at day 14 post-infection by magnetic bead separation and stimulated immediately ex vivo with PMA + ionomycin (50 ng/mL and 1 µM, respectively) in the presence of brefeldin A (5 μg/mL) for 5 h. Cells were stained with CD4 and ɣδ TCR antibodies, and subsequently fixed and permeabilized to evaluate IFN-ɣ, IL-17A, and TNF production by intracellular cytokine staining, where **B** percent cytokine-positive cells and **C** flow plots are presented from three independent experiments (*p < 0.05; **p < 0.01; unpaired Student’s *t*-test)

Craniotomy infections are not effectively treated without surgical intervention because of biofilm formation [43]. This suggests the existence of a suppressive or maladaptive immune response to prevent biofilm clearance. A likely candidate was IL-10 given its expression in tissues during *S. aureus* craniotomy infection and well-characterized effects on dampening proinflammatory activity in numerous leukocyte populations [10, 14, 20]. IL-10 production was most robust in granulocytes that invaded the brain and galea at days 7 and 14 post-infection, whereas expression in monocytes and microglia was lower (Fig. 1). Separation of granulocytes into G-MDSCs and PMNs revealed that G-MDSCs were the main source of IL-10 in the brain and galea at day 7 post-infection, which transitioned to equivalent production in both granulocyte populations by day 14 (Additional file 1: Fig. S3). Bacterial burden was significantly reduced in the brain and galea of IL-10 KO mice at day 14 post-infection compared to WT animals (Fig. 2A, B, respectively). The bone flap was less affected by IL-10 loss (Fig. 2C), which represents the biofilm nidus where bacteria adopt a more metabolically dormant state [5, 44] making them less susceptible to immune-mediated attack. Reduced bacterial burden in the brains of IL-10 KO mice at day 14 post-infection was associated with a significant decrease in G-MDSC infiltrates (Fig. 2A), a pathologically activated PMN population that inhibits T cell activation, macrophage proinflammatory activity, and PMN bactericidal activity [11, 45]. Monocyte recruitment to the brain was unaffected at this time point, but was significantly increased at an earlier interval (day 7 post-infection) in IL-10 KO animals that preceded the reduction in bacterial burden (Fig. 2A). Both ɣδ and CD4^+^ T cell infiltrates were significantly increased in the brains of IL-10 KO mice at days 7 and 14 post-infection, respectively (Fig. 3A), which when combined with the significant reduction in G-MDSCs suggests a transition in the inflammatory milieu in the absence of IL-10. In contrast to the brain, leukocyte infiltrates in the galea and bone flap were not significantly affected by IL-10 loss (Fig. 2B, C). Similar trends in leukocyte influx and microglial abundance were evident with absolute cell counts in each location (Additional file 1: Fig. S4).

**Fig. 4:**
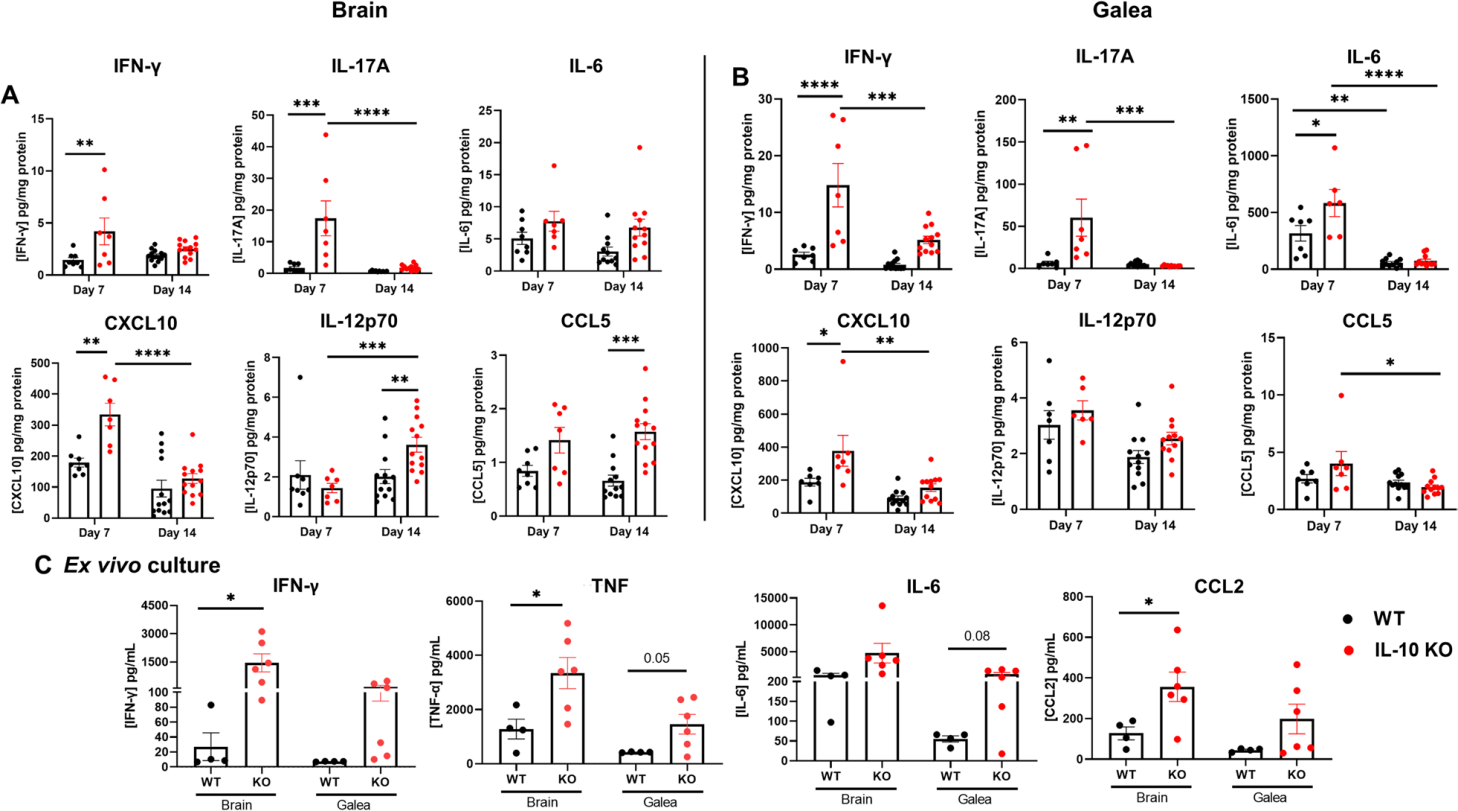
IL-10 loss results in increased cytokine and chemokine expression. IL-10 knockout (KO) and wild type (WT) mice were euthanized at day 7 (*n* = 8 WT and 7 KO) or day 14 (*n* = 13 WT and 13 KO) following *S. aureus* craniotomy infection, whereupon inflammatory mediator expression in the **A** brain and **B** galea was determined using Milliplex multi-analyte bead arrays (data compiled from 3 independent experiments). **C** Total leukocytes recovered from the brain at day 14 post-infection (*n* = 4 WT and *n* = 6 KO) were cultured overnight, whereupon inflammatory mediator production was quantified in conditioned medium by CBA (results combined from 3 independent experiments). Significant differences between IL-10 KO and WT mice were determined by **A** and **B** two-way ANOVA with Tukey’s correction and **C** unpaired Student’s *t*-test and are denoted by asterisks (*p < 0.05; **p < 0.01; ***p < 0.001; ****p < 0.0001)

Owing to the differential lymphocyte recruitment observed in IL-10 KO mice, we next investigated the effect of IL-10 on T cell cytokine production. A heightened proinflammatory signature was revealed by significant increases in the frequency of TNF and IFN-γ producing CD4^+^ T cells and TNF-positive γδ T cells in the brains of IL-10 KO mice at day 14 (Fig. 3B, C), which coincided with reduced bacterial burden (Fig. 2A). To further explore how IL-10 loss affects the inflammatory milieu during *S. aureus* craniotomy infection, inflammatory mediator expression was assessed in tissue homogenates of WT and IL-10 KO mice in addition to leukocytes recovered from infected tissues. Quantification of cytokines/chemokines in brain and galea homogenates revealed elevated IFN-γ, IL-17, IL-6, CXCL10 (IP-10), IL-12p70, and CCL5 (RANTES) in IL-10 KO compared to WT mice (Fig. 4A, B). In general, these inflammatory mediators were more dramatically increased in IL-10 KO animals at day 7 post-infection, preceding the significant reduction in bacterial burden observed at day 14 in the brain and galea (Fig. 2A, B). Interestingly, the increases in CXCL10 and CCL5 as well as IFN-γ and IL-17 in brain tissues of IL-10 KO mice may support, in part, the enhanced T cell recruitment and proinflammatory cytokine production by lymphocytes, respectively, in the IL-10 KO brain. Leukocytes cultured immediately ex vivo from infected IL-10 KO animals produced significantly higher levels of IFN-γ, TNF, and CCL2 compared to cells from WT mice (Fig. 4C). Given the increases in lymphocyte IFN-ɣ and TNF production in IL-10 KO animals, CD4^+^ or γδ T cells were depleted to determine if they were responsible for the reduction in *S. aureus* burden in IL-10 KO mice. Bacterial abundance in IL-10 KO animals remained unchanged following either CD4 or γδ T cell depletion (Additional file 1: Figs. S5 and S6, respectively), suggesting that neither lymphocyte population alone was responsible for lowering *S. aureus* burden. Collectively, these results demonstrate the establishment of a heightened proinflammatory response in the absence of IL-10 that is better capable of combating *S. aureus* infection in the brain and galea.

### IL-10 production by granulocytes is important for preventing *S. aureus* clearance in the galea during craniotomy infection

Since our findings revealed robust IL-10 production in granulocytes with less expression in microglia and monocytes (Fig. 1), we targeted these cell types by generating *Mrp8*^*Cre*^*IL-10*^* fl/fl*^ and *CX3CR1*^*Cre*^*IL-10*^* fl/fl*^ mice, respectively, to determine the critical source of IL-10 during craniotomy infection, with WT littermates as controls. For *CX3CR1*^*Cre*^*IL-10*^* fl/fl*^ mice, IL-10 deletion was confirmed by increased YFP expression in microglia and monocytes and lack of IL-10 production in the brain (Additional file 1: Fig. S7A, B). No dramatic changes were seen with respect to bacterial burden, immune cell infiltrates, or microglial abundance in *CX3CR1*^*Cre*^*IL-10*^* fl/fl*^ animals (Additional file 1: Fig. S7C, D). However, several chemokines responsible for PMN (CXCL1) and T cell (CXCL9, CXCL10, and CCL2) recruitment were significantly increased in the brains of *CX3CR1*^*Cre*^*IL-10*^* fl/fl*^ mice (Additional file 1: Fig. S7E). Of note, these changes only manifested in the brain where resident microglia and most monocyte/macrophage infiltrates are found, and not in the galea that is dominated by granulocyte recruitment. This reveals a footprint for IL-10 action in the brain; however, this was not sufficient to impact *S. aureus* burden (Additional file 1: Fig. S7C).

**Fig. 5:**
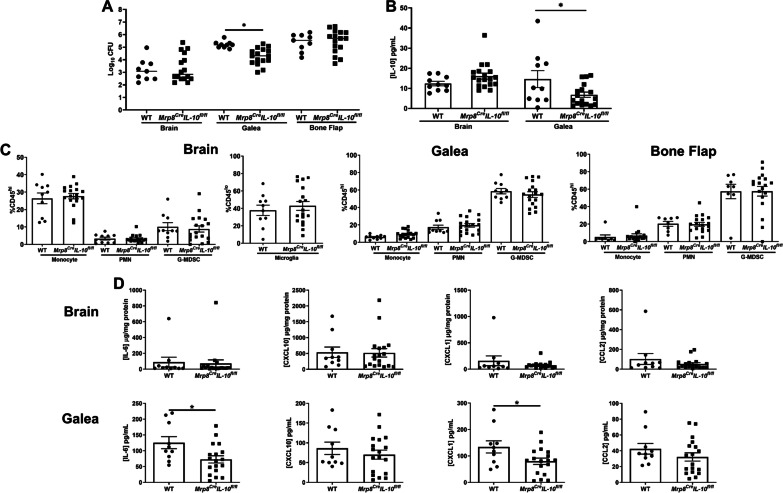
Granulocyte IL-10 production is important for promoting *S. aureus* persistence in the galea during craniotomy infection. *Mrp8*^*Cre*^*IL-10*^* fl/fl*^ (*n* = 15–20) and wild type (WT) littermates (*n* = 9–10) were euthanized at day 14 following *S. aureus* craniotomy infection, whereupon **A** bacterial burden, **B** IL-10 production, **C** monocyte, neutrophil (PMN), and granulocytic myeloid-derived suppressor cell (G-MDSC), infiltrates in the brain in addition to resident microglia, galea, and bone flap and **D** cytokine/chemokine expression was quantified in the brain and galea by Milliplex multi-analyte bead arrays. Data are combined from three independent experiments (*p < 0.05; unpaired Student’s *t*-test)

**Fig. 6:**
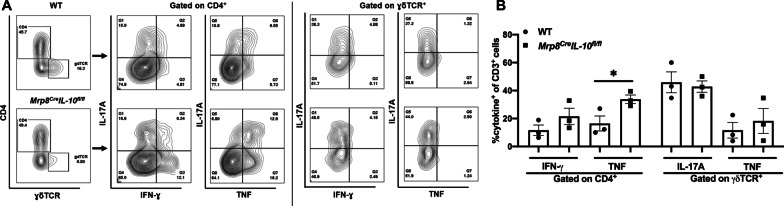
IL-10 loss in granulocytes leads to heightened cytokine production by CD4^+^ and T cells. CD3^+^ cells were isolated from the brains of *Mrp8*^*Cre*^*IL-10*^* fl/fl*^ and wild type (WT) littermates at day 14 post-infection by magnetic bead separation and stimulated immediately ex vivo with PMA + ionomycin (50 ng/mL and 1 µM, respectively) in the presence of brefeldin A (5 μg/mL) for 5 h. Cells were stained with CD4 and ɣδ TCR antibodies, and subsequently fixed and permeabilized to evaluate IFN-γ, IL-17A, and TNF production by intracellular cytokine staining. Data are combined from three independent experiments and presented as **A** flow plots and **B** percent cytokine-positive cells (*p < 0.05; unpaired Student’s *t*-test)

Since PMNs and G-MDSCs have been reported to produce IL-10 [32, 46–48], which was corroborated by our intracellular cytokine staining (Fig. 1 and Additional file 1: Fig. S3), we next examined craniotomy infection in *Mrp8*^*Cre*^*IL-10*^* fl/fl*^ mice with WT littermates as controls. In *Mrp8*^*Cre*^*IL-10*^* fl/fl*^ mice, IL-10 targeting was confirmed by YFP expression in PMNs and G-MDSCs (Additional file 1: Fig. S8) along with a significant decrease in IL-10 production in the galea, which translated into reduced bacterial burden selectively in this compartment (Fig. 5A, B). Although leukocyte recruitment and microglial abundance was similar between *Mrp8*^*Cre*^*IL-10*^* fl/fl*^ and WT mice (Fig. 5C), significant reductions in IL-6 and CXCL1 production were detected in the galea of *Mrp8*^*Cre*^*IL-10*^* fl/fl*^ animals (Fig. 5D). In contrast, no differences in IL-10 production, bacterial burden, or inflammatory mediator production were observed in the brain of *Mrp8*^*Cre*^*IL-10*^* fl/fl*^ mice compared to WT littermates (Fig. 5). The restriction of phenotypes to the galea of *Mrp8*^*Cre*^*IL-10*^* fl/fl*^ mice corroborates the preferential recruitment of granulocyte infiltrates in this compartment vs. the brain during craniotomy infection [10, 11]. Given the increased cytokine production by CD4^+^ and ɣδ T cells in IL-10 KO mice, we next examined whether IL-10 loss in granulocytes affected cytokine levels in either lymphocyte population. TNF expression was significantly increased in CD4^+^ T cells recovered from the brain of *Mrp8*^*Cre*^*IL-10*^* fl/fl*^ mice (Fig. 6), similar to IL-10 KO animals (Fig. 3B). Collectively these observations suggest that granulocyte-derived IL-10 regulates select proinflammatory responses to promote *S. aureus* persistence in the galea during craniotomy infection. Although IL-10 is expressed by microglia and monocytes during infection (Fig. 1), this source appears to have little impact on *S. aureus* survival (Additional file 1: Fig. S7C).

**Fig. 7:**
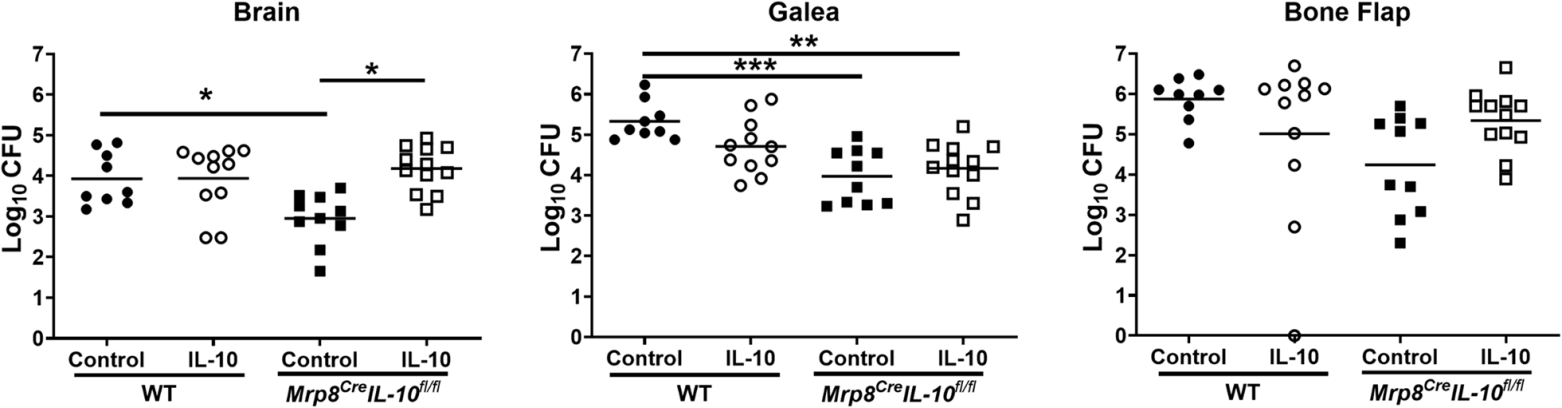
Exogenous IL-10 inhibits *S. aureus* clearance in *Mrp8*^*Cre*^*IL-10*^* fl/fl*^ mice. *Mrp8*^*Cre*^*IL-10*^* fl/fl*^ mice and WT littermates received IL-10 containing (*n* = 11 WT and 12 Cre) or control (vehicle; *n* = 9 WT and 10 Cre) microparticles applied at the dorsal and ventral aspects of the bone flap on the day of *S. aureus* infection and were euthanized at day 14 to quantify bacterial burden in the brain, galea, and bone flap (*p < 0.05; **p < 0.01; ***p < 0.001; one-way ANOVA)

To confirm the action of IL-10 in *Mrp8*^*Cre*^*IL-10*^* fl/fl*^ mice, IL-10 containing microparticles were administered at the time of infection to provide a continual source of the cytokine (Additional file 1: Fig. S2). IL-10 microparticles significantly increased *S. aureus* burden in the brain of *Mrp8*^*Cre*^*IL-10*^* fl/fl*^ animals compared to empty microparticles, with bacterial abundance reaching that of WT mice (Fig. 7). Similar trends were observed in the galea and bone flap but did not reach statistical significance. Interestingly, the addition of exogenous IL-10 did not exacerbate *S. aureus* infection in WT littermates (Fig. 7). Collectively, these findings establish the importance of granulocyte-derived IL-10 in preventing *S. aureus* clearance from the galea during craniotomy infection.

### G-MDSC-derived IL-10 is critical for inhibiting PMN bactericidal activity and TNF production

**Fig. 8:**
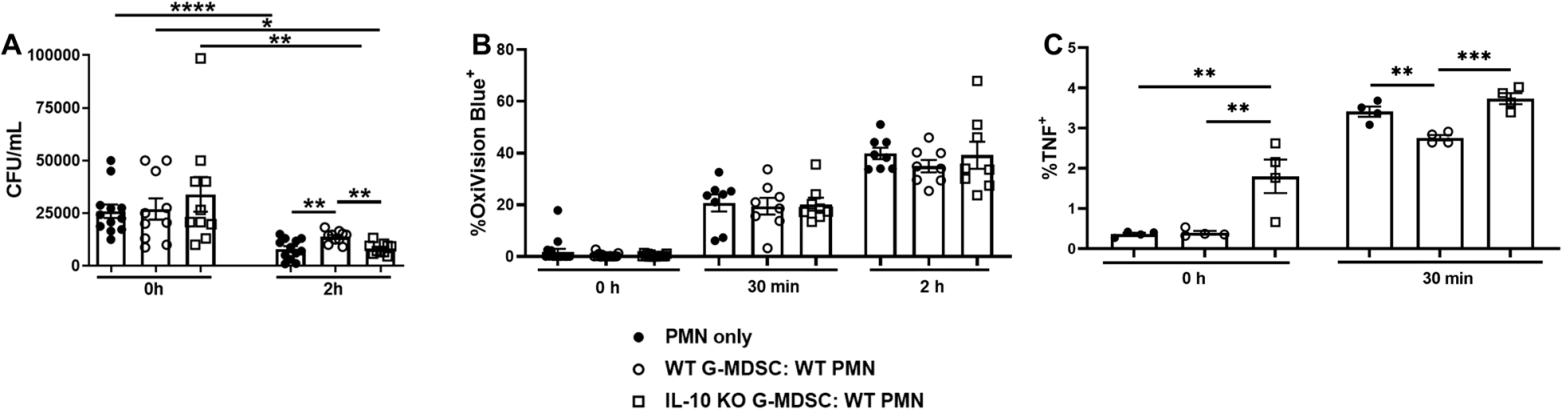
IL-10 production by G-MDSCs is critical for inhibiting PMN bactericidal activity and TNF production. PMNs were exposed to live *S. aureus* USA300 LAC for 2 h at an MOI of 10:1 (bacteria:cell) in the presence of IL-10 KO or WT G-MDSCs separated by Transwell inserts to evaluate the functional importance of G-MDSC-derived IL-10 on **A** PMN killing of *S. aureus* by gentamicin protection assays (*n* = 10–12 biological replicates), **B** H_2_O_2_ production using OxiVision dye (*n* = 8 biological replicates), and **C** TNF production by intracellular staining (*n* = 4 biological replicates) (*p < 0.05; **, *p* < 0.01; ***p < 0.001; ****p < 0.0001; One-way ANOVA for each time point)

Finally, we explored the possibility that IL-10 production by G-MDSCs was one mechanism responsible for *S. aureus* persistence during craniotomy infection, given that *Mrp8*^*Cre*^*IL-10*^* fl/fl*^ mice had lower bacterial burden in the galea where G-MDSCs are abundant. G-MDSCs from WT animals inhibited PMN bactericidal activity at 2 h in agreement with our previous findings [11], which was not observed with G-MDSCs from IL-10 KO mice (Fig. 8A). This finding demonstrates that the ability of G-MDSCs to attenuate PMN killing of *S. aureus* is IL-10-dependent.

To further investigate how G-MDSC-derived IL-10 may affect PMN function, H_2_O_2_ and cytokine production in response to live *S. aureus* was examined. As expected, H_2_O_2_ production was increased in PMNs within 30 min following *S. aureus* exposure; however, no significant differences in H_2_O_2_ levels were observed when PMNs were co-cultured with either WT or IL-10 KO G-MDSCs (Fig. 8B). TNF production was significantly induced by PMNs in response to *S. aureus* at 30 min and was attenuated by G-MDSC-derived IL-10, since TNF expression was significantly reduced following co-culture with WT G-MDSCs, which was restored in response to G-MDSCs from IL-10 KO mice (Fig. 8C).

Finally, cell intrinsic actions of IL-10 on PMN activity were examined using PMNs isolated from *Mrp8*^*Cre*^*IL-10*^* fl/fl*^ mice with cells from *CX3CR1*^*Cre*^*IL-10*^* fl/fl*^ animals as a comparator. No significant differences in *S. aureus* intracellular burden or cytokine production were observed between PMNs from either conditional KO line or WT littermates (Additional file 1: Fig. S9), suggesting that cell-extrinsic actions of IL-10 are critical for regulating PMN effector function, which our studies show is mediated, in part, by the inhibitory action of G-MDSCs.

## Discussion

The current study implicates a pathological role for IL-10 in attenuating proinflammatory pathways to prevent biofilm clearance during *S. aureus* craniotomy infection, which is supported by several lines of evidence. First, IL-10 production by G-MDSCs was important for attenuating *S. aureus* killing and TNF expression by PMNs. Second, IL-10 was pivotal for limiting CD4^+^ and γδ T cell recruitment and activation, as both lymphocyte infiltrates were increased in the brain of IL-10 KO mice and produced heightened levels of IFN-ɣ and TNF that coincided with decreased bacterial burden. Third, IL-10 was critical for diminishing the production of IL-17, IFN-ɣ, and the IFN-ɣ-induced chemokine CXCL10 in the infected brain and galea, since all were significantly elevated in tissues from IL-10 KO animals. Cross-regulation of IFN-ɣ and IL-10 has been reported in macrophages and several disease models [49–54], where IL-10 production overrides IFN-ɣ leading to chronic infection [55, 56]. To determine whether the increases in CD4^+^ and ɣδ T cell recruitment in the brain of IL-10 KO mice were responsible for the lower bacterial burden in these animals, both populations were depleted. However, removing either population had no significant impact on infectious burdens in IL-10 KO animals, suggesting either redundancies in lymphocyte action and/or the involvement of different effector cell type(s) that remain to be determined. Another outstanding question is whether biofilm properties are altered in IL-10 KO mice. Because bacterial abundance was decreased in IL-10 KO animals, it is possible that the biofilm transitioned to a more planktonic state. This could be pursued in future studies with systemic antibiotic treatment of IL-10 KO mice, since planktonic organisms are more susceptible to antibiotics compared to biofilm, along with SEM to evaluate biofilm morphology on the bone flap. However, this was beyond the scope of the current report.

As IL-10 KO mice established an important role for the cytokine in promoting *S. aureus* persistence during craniotomy infection, as evident by significantly lower bacterial burden in the brain and galea at later stages of infection, this led us to evaluate the possible cellular sources of IL-10. Since our findings revealed that granulocytes were a major source of IL-10 during *S. aureus* craniotomy infection with less production by microglia and monocytes, we generated *Mrp8*^*Cre*^*IL-10*^* fl/fl*^ and *CX3CR1*^*Cre*^*IL-10*^* fl/fl*^ mice to target each population, respectively. Although some inflammatory mediators were altered in *CX3CR1*^*Cre*^*IL-10*^* fl/fl*^ animals, this did not translate into less infectious burden, suggesting a minor role for microglial/monocyte-derived IL-10 in dictating infection chronicity. Surprisingly, only *Mrp8*^*Cre*^*IL-10*^* fl/fl*^ mice displayed reduced *S. aureus* abundance that coincided with decreased expression of several mediators in the galea. The fact that phenotypes only manifested in the galea of *Mrp8*^*Cre*^*IL-10*^* fl/fl*^ animals agrees with the finding that granulocyte infiltrates dominate this compartment compared to the brain. However, the combined action of IL-10 production by PMNs/G-MDSCs and microglia/monocytes cannot be disregarded based on the more dramatic phenotype of IL-10 KO mice that was not recapitulated with either conditional KO line. A potential role for astrocyte- or T cell-derived IL-10 [11, 20, 57] may also contribute to promoting *S. aureus* craniotomy infection. However, T cells recovered from the brain of infected WT mice displayed limited IL-10 production, suggesting they are not likely a critical source of the cytokine. It was interesting that cytokine/chemokine concentrations were lower in the galea of *Mrp8*^*Cre*^*IL-10*^* fl/fl*^ mice that had better outcomes (i.e., reduced bacterial burden), whereas increased mediator production coincided with improved bacterial clearance in IL-10 KO animals. The most plausible explanation for this finding is differences between the mouse models, since the cytokine is completely absent in IL-10 KO mice, whereas IL-10 deletion is only targeted to granulocytes in *Mrp8*^*Cre*^*IL-10*^* fl/fl*^ animals and other cell types can still produce the cytokine. Since granulocytes are the most abundant infiltrate in the galea, and are the major source of IL-10, this is likely why a phenotype manifested there. In contrast, fewer granulocytes infiltrate the brain, where IL-10 is still produced by microglia and monocytes, albeit at lower levels, such that phenotypes were not evident in the brain. Furthermore, the proportions of immune cell infiltrates and mediators that were significantly altered between IL-10 KO vs. *Mrp8*^*Cre*^*IL-10*^* fl/fl*^ mice were distinct, making direct comparisons difficult.

Another question that remains is the target population of IL-10 action. IL-10 acts via a receptor complex composed of a high affinity IL-10Rα that is largely restricted to leukocytes and IL-10Rβ which is more ubiquitous. The downstream signaling pathway emanating from the receptor complex initiates a cascade of anti-inflammatory responses mediated through STAT3 signaling [58]. Regarding *S. aureus* infection, PMNs are a major source of IL-10 during *S. aureus*-induced sepsis and accelerate disease progression, which is exacerbated by IFN-ɣ [46, 59]. Myeloid cells have been shown to produce IL-10 and facilitate *S. aureus* persistence in the nares in an lL-27-dependent manner [60]. Furthermore, *S. aureus* induces IL-10 production from microglia [61] and several *S. aureus* PAMPs can trigger IL-10 release from monocytes and macrophages [62] in a TLR2-dependent manner. In terms of effector functions, IL-10 has been shown to attenuate macrophage proinflammatory activity [32] and Th1, Th17, and ɣδ T cell activation [31, 60, 63] in response to *S. aureus*. The latter findings align with our observations, since T cell responses were increased in IL-10 KO mice, typified by elevated IFN-ɣ and TNF production by CD4^+^ and ɣδ T cells. Saxton et al. recently showed that expression levels of the low-affinity IL-10Rβ subunit are critical for dictating cell type-dependent effects of IL-10, namely inhibition of monocyte/macrophage proinflammatory activity vs. stimulating inflammatory CD8^+^ T cells, providing a molecular mechanism to explain the anti- vs. pro-inflammatory attributes of IL-10, respectively [64]. This could be another explanation for the more limited phenotypes of IL-10 conditional KO compared to IL-10 KO mice because cells that express high levels of IL-10Rβ would remain responsive to the lower cytokine levels present in IL-10 conditional KO mice. It would be interesting to investigate IL-10Rβ expression on various cell populations during *S. aureus* craniotomy infection and whether levels fluctuate over time to determine how this coincides with the kinetics of IL-10 action identified in this study.

An enigmatic relationship exists for how leukocytes regulate *S. aureus* persistence vs. resolution during planktonic and biofilm infections, which is likely influenced by the tissue microenvironment and bacterial growth state. For example, planktonic *S. aureus* infection (i.e., abscesses, skin and soft tissue infection) typically elicits a robust innate immune response, dominated by PMNs and macrophages, along with innate lymphoid (ɣδ T cells) and adaptive (Th1 and Th17) populations [65, 66]. The collective action of these cell types typically leads to infection resolution, although protective memory responses to *S. aureus* are inefficient and recurrent infections are common [67–69]. Paradoxically, the same leukocyte populations are present during *S. aureus* craniotomy infection, yet they are not capable of clearing the biofilm [10, 11, 30, 36]. The reasons responsible for these differences between *S. aureus* biofilm vs. planktonic infection remain unclear; however, this may be attributed to the compartmentalized recruitment of leukocytes during craniotomy infection (where granulocytes preferentially traffic to the galea, whereas monocytes/macrophages and T cell populations infiltrate the brain) and programming by distinct microenvironmental signals [11, 30, 36], whereas these leukocyte populations converge in the same general location during planktonic *S. aureus* infections. However, the most likely driver for programming a non-productive immune response during craniotomy infection is the biofilm itself [70, 71]. As previously discussed, biofilm represents a unique growth state compared to planktonic bacteria and *S. aureus* biofilm polarizes macrophages towards an anti-inflammatory state and leads to preferential G-MDSC recruitment [38, 45, 72]. Furthermore, *S. aureus* biofilm growth is associated with metabolites, such as lactate and formate, that interfere with innate immune responses that would typically clear planktonic infection [33, 73]. However, most of this information originates from models of biofilm infection in the periphery; therefore, much work remains to be done to understand the mechanisms responsible for driving a maladaptive immune response during craniotomy biofilm infection, both from the host and pathogen perspectives.

Although granulocyte IL-10 production was important for promoting bacterial persistence in the galea during *S. aureus* craniotomy infection, our prior report revealed that granulocyte depletion with anti-Ly6G resulted in widespread increases in bacterial burden in all tissue compartments [11]. This reflects a double-edged sword for granulocytes in biofilm pathogenesis, where PMNs are critical for pathogen containment, yet they can also serve as a source of IL-10 along with G-MDSCs to counteract the proinflammatory action of other innate and adaptive immune cells. It would be interesting to determine whether granulocyte depletion with anti-Ly6G at later stages of craniotomy infection would promote bacterial clearance given the known immunosuppressive role of IL-10 at this interval. However, a more likely scenario is that IL-10 loss alleviates a negative signal on granulocyte activation, allowing them to better neutralize *S. aureus*, which accounts for reduced bacterial abundance in the galea of *Mrp8*^*Cre*^*IL-10*^* fl/fl*^ animals. Further evidence from this study revealed the cross-regulatory role of granulocyte-derived IL-10 on proinflammatory cytokine production, since CD4^+^ T cells infiltrating the brain of *Mrp8*^*Cre*^*IL-10*^* fl/fl*^ mice produced higher levels of TNF. Several reports have shown that PMNs [46, 47, 59, 74–76] and G-MDSCs [32, 48, 77] are a major source of IL-10 in agreement with our findings. A prior study reported the presence of a PMN population that expresses IL-10 and polarizes macrophages to an anti-inflammatory phenotype in a SCID mouse model of severe inflammatory response syndrome caused by *S. aureus* [78]. These PMNs may represent G-MDSCs, but at the time this possibility was not pursued.

Collectively, this work has demonstrated an important role for IL-10 in promoting *S. aureus* persistence during craniotomy infection. This is likely mediated, in part, by the inhibitory effects of G-MDSC-derived IL-10 on PMN bactericidal activity and TNF production and overall dampening of proinflammatory cytokine production by T cells, particularly IFN-ɣ and TNF. These findings highlight the complex role of IL-10 during *S. aureus* biofilm infection in the CNS.

## Supplementary Information

**Additional file 1: Figure S1.** Gating strategy to quantify immune populations in the brain and galea following *S. aureus* craniotomy infection. **Figure S2.** Characterization of IL-10 containing microparticles. **Figure S3.** Absolute numbers of IL-10 producing G-MDSCs and PMNs during *S. aureus* craniotomy infection. **Figure S4.** Absolute numbers of immune cell infiltrates in WT and IL-10 KO mice during craniotomy infection. **Figure S5.** CD4+ T cells are not critical for *S. aureus *containment during craniotomy infection in IL-10 knockout mice. **Figure S6.** ɣδ T cells do not influence bacterial growth during *S. aureus* craniotomy infection. Figure S7. IL-10 deletion in monocytes and microglia has minimal effects on *S. aureus* craniotomy infection. **Figure S8.** Characterization of IL-10 deletion from G-MDSCs and PMNs in Mrp8CreIL-10fl/fl mice. **Figure S9.** *S. aureus* intracellular burden and cytokine production are not affected in PMNs from Mrp8CreIL-10fl/fl or CX3CR1CreIL-10fl/fl mice.

## Data Availability

All data and mice included in this manuscript are available upon reasonable request.

## Abbreviations

CNS: Central nervous system
CFU: Colony forming units
cKO: Conditional knockout
CX3CR1: Fractalkine receptor
ɣδ T cell: Gamma-delta T cell
G-MDSC: Granulocytic myeloid-derived suppressor cell
IL-10: Interleukin-10
IFN-ɣ: Interferon-gamma
KO: Knockout
Mrp8: Myeloid-related protein 8
PBS: Phosphate buffered saline
PMN: Polymorphonuclear neutrophil
STAT3: Signal transducer and activator of transcription 3
TLR2: Toll-like receptor 2
TNF: Tumor necrosis factor
WT: Wild type
YFP: Yellow fluorescent protein
