# Supplementary material for: IL-10 production by granulocytes promotes Staphylococcus aureus craniotomy infection
Source: J Neuroinflammation. 2023 May 13;20:114. doi: 10.1186/s12974-023-02798-7 (PMC10183138; doi:10.1186/s12974-023-02798-7)
Supplement: Supplementary file 1 — Additional file 1: Figure S1. Gating strategy to quantify immune populations in the brain and galea following S. aureus craniotomy infection. Figure S2. Characterization of IL-10 containing microparticles. Figure S3. Absolute numbers of IL-10 producing G-MDSCs and PMNs during S. aureus craniotomy infection. Figure S4. Absolute numbers of immune cell infiltrates in WT and IL-10 KO mice during craniotomy infection. Figure S5. CD4+ T cells are not critical for S. aureus containment during craniotomy infection in IL-10 knockout mice. Figure S6. ɣδ T cells do not influence bacterial growth during S. aureus craniotomy infection. Figure S7. IL-10 deletion in monocytes and microglia has minimal effects on S. aureus craniotomy infection. Figure S8. Characterization of IL-10 deletion from G-MDSCs and PMNs in Mrp8CreIL-10fl/fl mice. Figure S9. S. aureus intracellular burden and cytokine production are not affected in PMNs from Mrp8CreIL-10fl/fl or CX3CR1CreIL-10fl/fl mice. [file 12974_2023_2798_MOESM1_ESM.pdf]

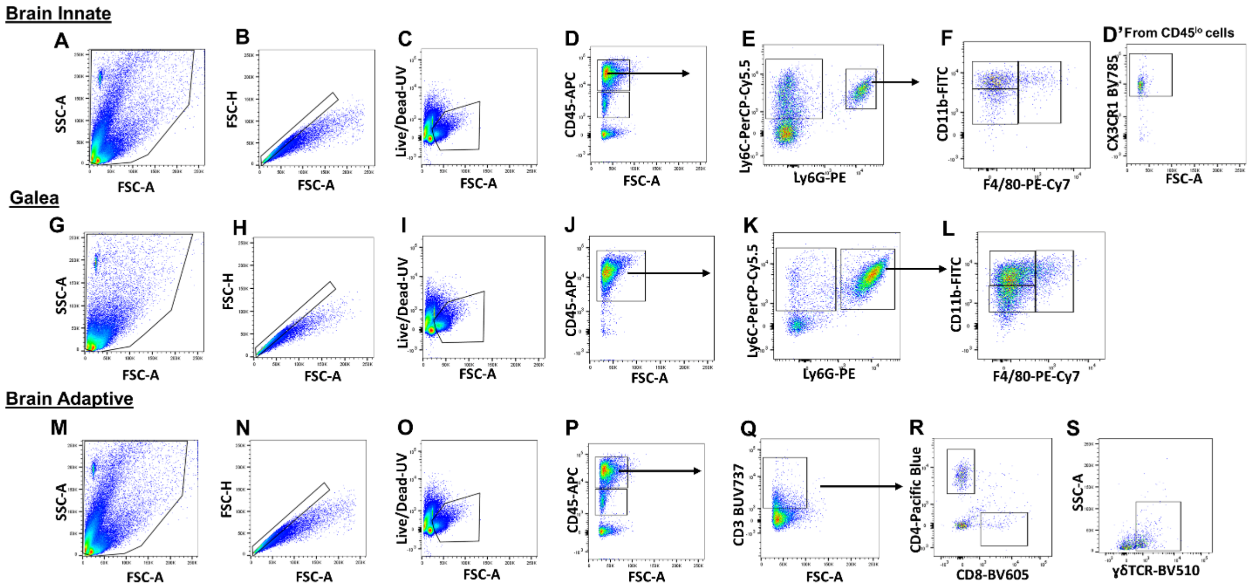

Additional file 1: Fig. S1. Gating strategy to quantify immune populations in the brain and galea following *S. aureus* craniotomy infection. From the (**A**, **G, M**) total events, (**B**, **H, N**) single cells were gated using FSC-A vs. FSC-H, followed by (**C**, **I, O**) exclusion of dead cells. For the innate immune panel, (**D, J**) live, CD45^{high} leukocytes were separated into (**E, K**) Ly6G^{-} Ly6C^{+} monocytes vs. Ly6G^{+}Ly6C^{+} cells, which were further identified as (**F, L**) G-MDSCs (CD11b^{high}Ly6G^{+}Ly6C^{+}F4/80^{-}) and neutrophils (CD11b^{low}Ly6G^{+}Ly6C^{+}F4/80^{-}) based on CD11b and F4/80 expression. Microglia were defined as (**D'**) CD45^{low} vs. FSC and CX3CR1^{+}. Adaptive immune cells were subjected to the same initial steps (**M**-**O**) as described for the innate immune panel, where (**P**) live, CD45^{high} leukocytes were separated into (**Q**) CD3^{+} cells that were gated on (**R**) CD4^{+} and CD8^{+} T cells and (**S**) ɣδ T cells (ɣδ TcR^{+}).

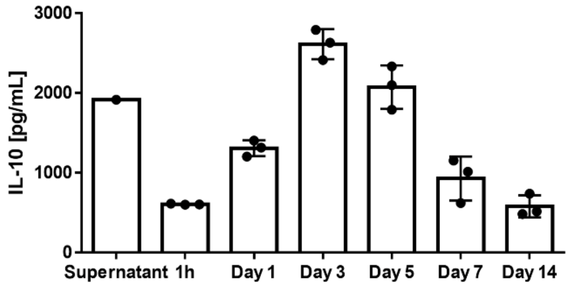

Additional file 1: Fig. S2. Characterization of IL-10 containing microparticles. Release kinetics of IL-10 loaded poly(lactide-co-glycolide) (PLGA) microparticles (n=3 per time point) over a 14-day period *in vitro* vs. non-encapsulated cytokine in the supernatant (n=1) following microparticle synthesis.

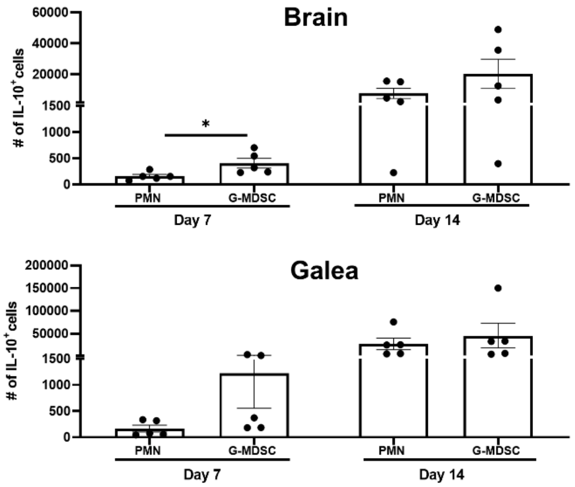

Additional file 1: Fig. S3. Absolute numbers of IL-10 producing G-MDSCs and PMNs during *S. aureus* craniotomy infection. Wild type mice (n=5/group) were sacrificed at days 7 and 14 following *S. aureus* craniotomy infection, whereupon the absolute numbers of IL-10^+ granulocytic myeloid-derived suppressor cells (G-MDSCs) and neutrophils (PMNs) in the brain and galea were determined using counting beads (*, $p < 0.05$; unpaired Student's *t*-test).

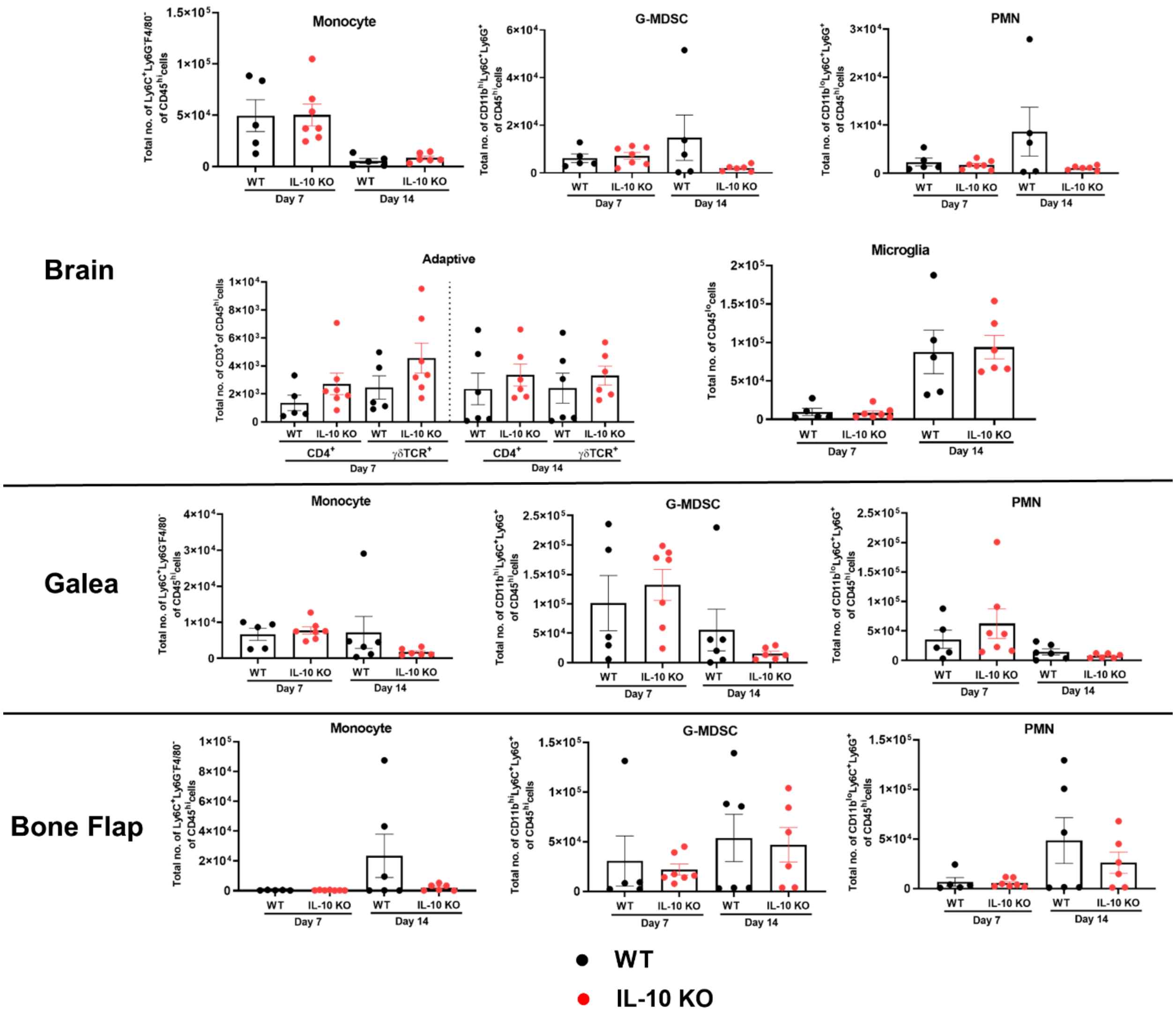

Additional file 1: Fig. S4. Absolute numbers of immune cell infiltrates in WT and IL-10 KO mice during craniotomy infection. Wild type (WT; n=5-6) and IL-10 knockout (KO; n=6-7) mice were sacrificed at days 7 and 14 following *S. aureus* craniotomy infection, whereupon the absolute numbers of resident microglia and leukocytes in the brain, galea, and bone flap were determined using counting beads.

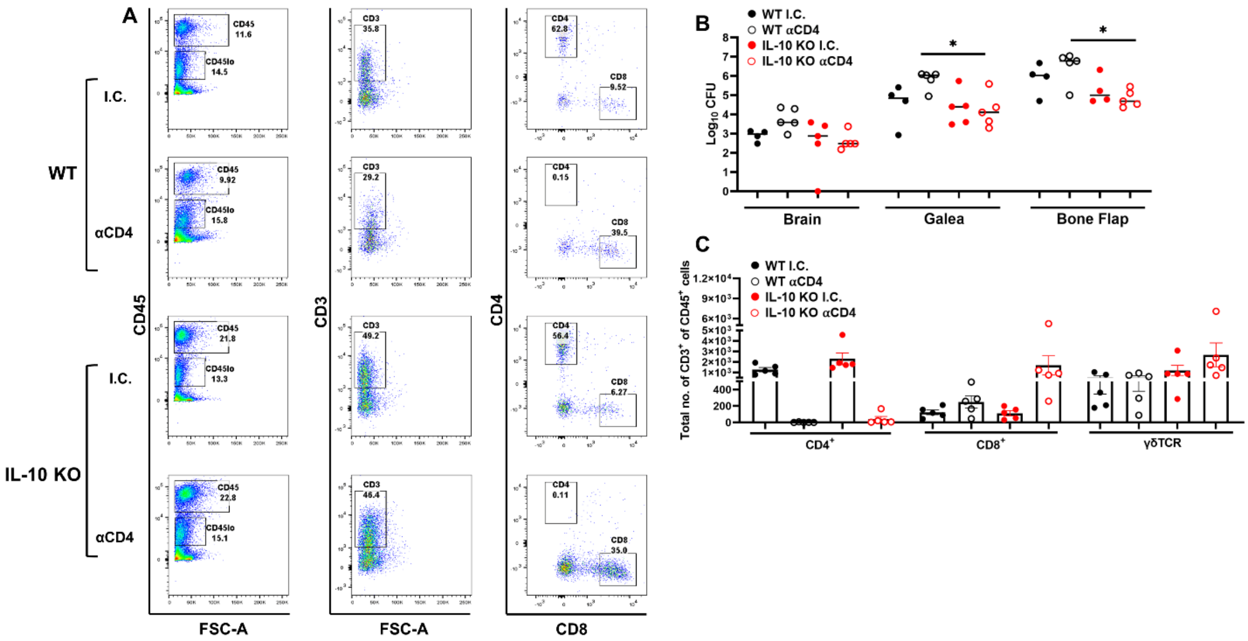

Additional file 1: Fig. S5. CD4 T cells are not critical for *S. aureus* containment during craniotomy infection in IL-10 knockout mice. Wild type (WT; n=4) and IL-10 knockout (KO; n=5) animals received anti-CD4 or isotype-matched control antibody (I.C.) beginning 3 days prior to *S. aureus* infection and every fourth day until sacrifice at day 14 post-infection, whereupon (**A**) $CD4^+$ T cell depletion in the brain and (**B**) bacterial burden in various CNS compartments were assessed. (**C**) Absolute number of leukocytes infiltrating the brain was quantified using counting beads via flow cytometry. Results are presented as the total number of $CD4^+$, $CD8^+$ or γδ T cells gated from live $CD3^+CD45^+$ cells (mean $\pm$ SEM). Significant differences were determined using a One-way ANOVA with Tukey's correction and are denoted by asterisks (*, $p < 0.05$).

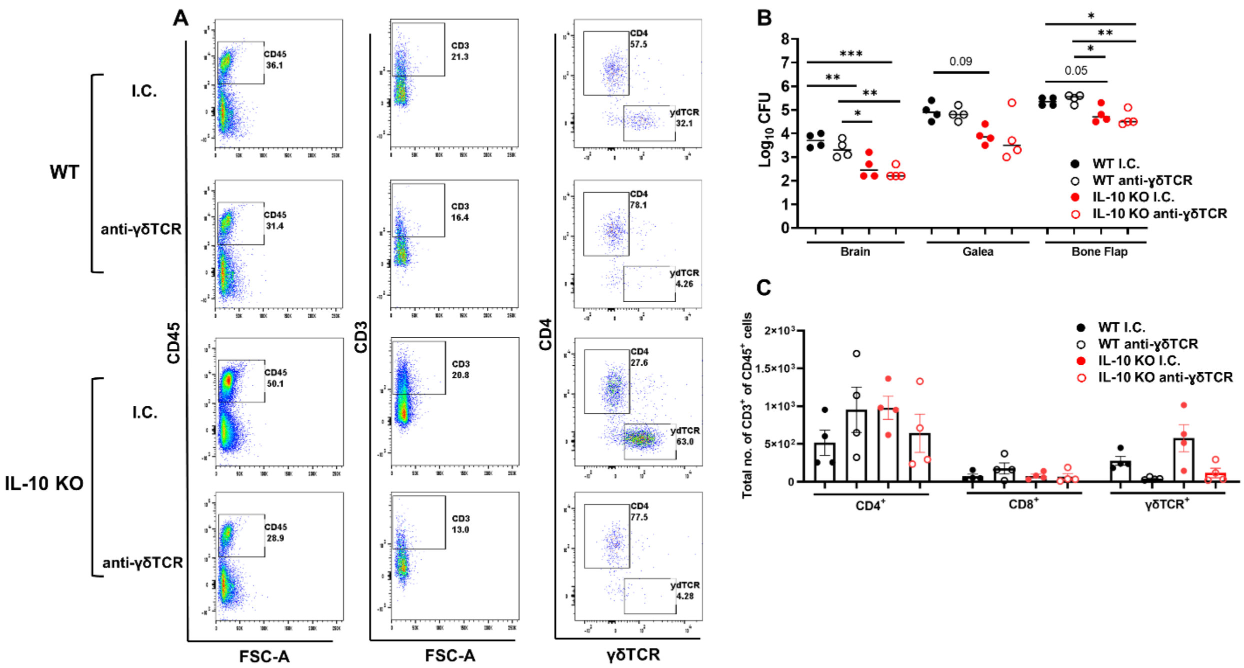

Additional file 1: Fig. S6. ɣδ T cells do not influence bacterial growth during *S. aureus* craniotomy infection. Wild type (WT) and IL-10 knockout (KO) animals (n=4/group) received anti-ɣδ TCR or isotype-matched control antibody (I.C.) beginning two days prior to *S. aureus* infection and every third day until sacrifice at day 14 post-infection, whereupon (**A**) ɣδ T cell depletion and (**B**) bacterial burden was assessed. (**C**) Absolute number of leukocytes infiltrating the brain was quantified using counting beads via flow cytometry. Results are presented as the total number of $CD4^+$, $CD8^+$ or ɣδ T cells gated from live $CD3^+CD45^+$ cells (mean $\pm$ SEM). Significant differences were determined using a One-way ANOVA with Tukey's correction and are denoted by asterisks (*, $p < 0.05$; **, $p < 0.01$; ***, $p < 0.001$).

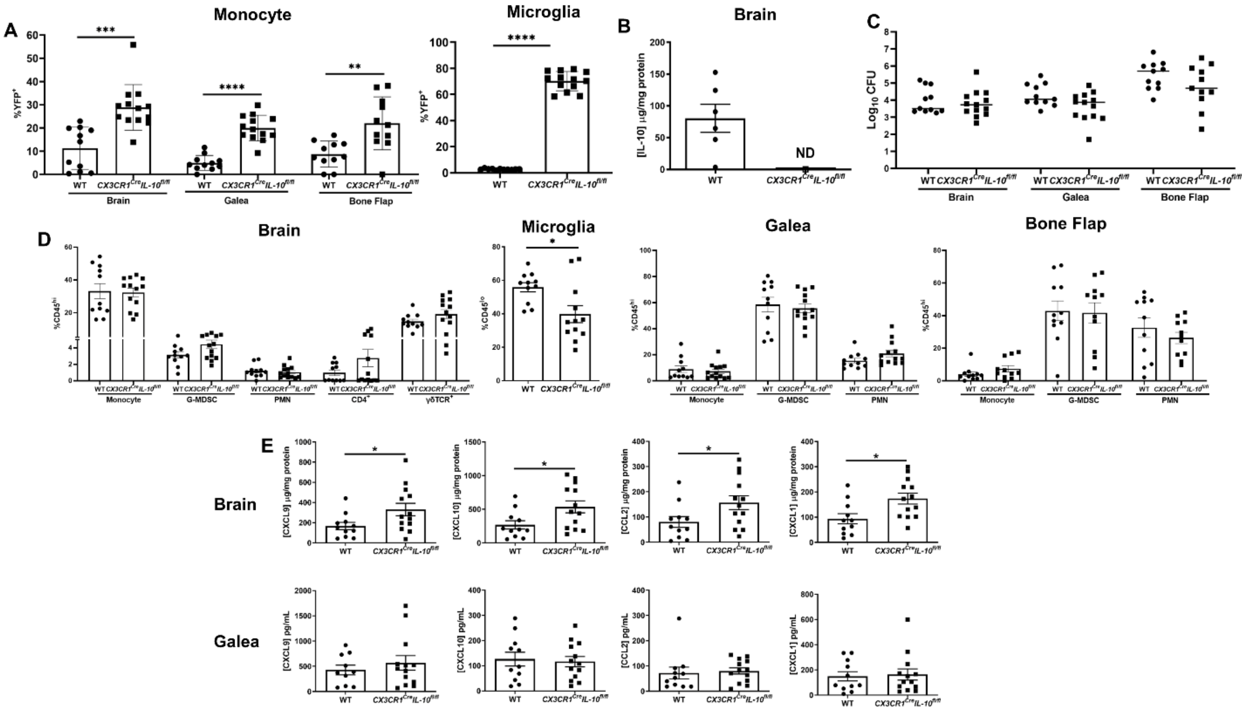

Additional file 1: Fig. S7. IL-10 deletion in monocytes and microglia has minimal effects on *S. aureus* craniotomy infection. (**A**) Percentage of YFP^+ microglia as well as monocytes in the brain, galea, and bone flap from *CX3CR1^{Cre}IL-10$^{fl/fl}$* mice (n=12-13) and WT littermates (n=11-13) at day 14 following *S. aureus* craniotomy infection. (**B**) Quantification of IL-10 levels in the brain by ELISA at day 14 post-infection (n=6 WT and 7 Cre). (**C**) Bacterial burden and (**D**) resident microglia and leukocyte infiltrates in the brain, galea, and bone flap, and (**E**) chemokine expression (n=11 WT and 13 Cre), was determined at day 14 after infection (*, $p < 0.05$; **, $p < 0.01$; ***, $p < 0.001$; ****, $p < 0.0001$; unpaired Student's *t*-test). ND, not detected.

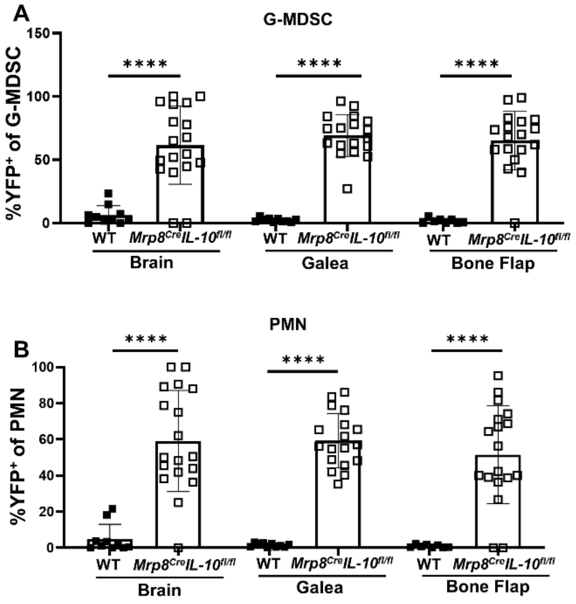

Additional file 1: Fig .S8. Characterization of IL-10 deletion from G-MDSCs and PMNs in *Mrp8^{Cre}IL-10$^{fl/fl}$* mice. Percentage of (**A**) YFP^{+} granulocytic myeloid-derived suppressor cells (G-MDSCs) and (**B**) neutrophils (PMNs) in the brain, galea, and bone flap of *Mrp8^{Cre}IL-10$^{fl/fl}$* mice (n=11-18) and WT littermates (n=8-15) at day 14 following *S. aureus* craniotomy infection ($p <$ 0.0001; unpaired Student's *t*-test).

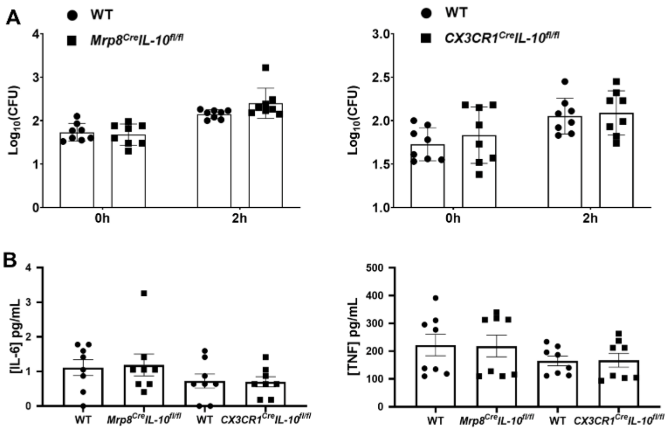

Additional file 1: Fig. S9. *S. aureus* intracellular burden and cytokine production are not affected in PMNs from $Mrp8^{Cre}IL\text{-}10^{fl/fl}$ or $CX3CR1^{Cre}IL\text{-}10^{fl/fl}$ mice. PMNs isolated from $Mrp8^{Cre}IL\text{-}10^{fl/fl}$ or $CX3CR1^{Cre}IL\text{-}10^{fl/fl}$ animals were exposed to live *S. aureus* USA300 LAC for 2 h at an MOI of 10:1 (bacteria:cell) to evaluate (**A**) *S. aureus* survival by gentamicin protection assays and (**B**) cytokine production by cytometric bead array (n=8 biological replicates/group combined from two independent experiments).
